# The Protean Forms of Tuberculosis

**Published:** 1898-07

**Authors:** 


### THE PROTEAN FORMS OF TUBERCULOSIS.

Thanks to bacteriologists and pathologists our ideas concerning many diseases are greatly changed from the opinions of the last generation. As a result of the development of these two sciences, the supposed relationship of many diseases, and their classification, have been modified to a considerable extent, and with two opposite effects. Distinct diseases like gonorrhœa, chancroid and syphilis, associated in origin, and frequently assumed to be related forms of the same disease, have been thoroughly differentiated. So, too, sarcoma and actinomycosis hominis, though symptomatically similar, and pathologically nearly identical, have revealed their distinguishing characteristics. On the other hand, apparently dissimilar affections, like psoas abscess and lupus have been shown to be only different manifestations of the same disease. For our knowledge of tuberculosis we owe much to these sciences. In the recognition of the cause, the symptoms and the diagnosis of these conditions, we have made great progress. Furthermore, the determination of the primary and secondary forms of the disease by the microscope and the needle has become possible, and often materially modifies the prognosis. In treatment we have progressed also, and yet we must frequently admit our helplessness. Surgery is able to do much more than ever before, and yet it has its limitations. From serum therapy we have had a degree of encouragement. In the absence of a more specific antitoxic remedy, preventive medicine offers our greatest resource against this hydra-headed disease.

